# Structure and Functioning of Acute Inpatient Psychiatric Units in Spain: Qualitative Study

**DOI:** 10.2196/26214

**Published:** 2021-04-07

**Authors:** Roberto Rodriguez-Jimenez, Iluminada Corripio, Ricardo Campos, Mario Páramo, Manuel Franco-Martin, Estefanía Segura, Sergio González, José Martínez-Raga

**Affiliations:** 1 Department of Psychiatry Instituto de Investigación Sanitaria Hospital 12 de Octubre (imas12) Madrid Spain; 2 Biomedical Research Networking Centre in Mental Health (CIBERSAM) Madrid Spain; 3 Faculty of Medicine Universidad Complutense de Madrid Madrid Spain; 4 Department of Psychiatry Hospital de la Santa Creu i Sant Pau Barcelona Spain; 5 Department of Psychiatry Hospital Clínico Universitario Lozano Blesa Zaragoza Spain; 6 Department of Psychiatry Hospital de Conxo Universidad de Santiago Santiago de Compostela Spain; 7 Department of Psychiatry Jefe del servicio de psiquiatría del Hospital Universitario Rio Hortega de Valladolid Valladolid Spain; 8 Department of Psychiatry Complejo Asistencial de Zamora Zamora Spain; 9 Department of Psychiatry Hospital General Universitario de Ciudad Real Ciudad Real Spain; 10 Department of Psychiatry Hospital Universitario Virgen Macarena Sevilla Spain; 11 Department of Psychiatry Faculty of Medicine University of Seville Sevilla Spain; 12 Department of Psychiatry Hospital Universitario Doctor Peset Valencia Spain

**Keywords:** acute inpatient psychiatric units, organization, resources, scorecard, Spain

## Abstract

**Background:**

As a consequence of the decentralization of health care provision to the different Regions (called Autonomous Communities) in Spain, different health care models and resources have been developed for psychiatric patients. It would be very useful to obtain comprehensive and comparative data on health care models, resources, and activity of acute inpatient psychiatric units (AIPUs) as a key part of mental health systems.

**Objective:**

The aim of this study was to determine the current state of AIPUs in Spain through a national scorecard that allows the current situation to be visualized in terms of resources, processes, and outputs.

**Methods:**

A 104-item online questionnaire was sent to all the AIPUs of the different Regions in Spain. It was divided into 11 sections, including data on the resources, processes, and outputs of the AIPUs plus general data, an indicator dashboard, and good practices.

**Results:**

The questionnaire was completed by 60.0% (117/195) of the AIPUs invited to participate. The information collected has allowed us to obtain a detailed snapshot of the current situation of AIPUs in Spain at the levels of infrastructure and material resources, staffing, organization and activity of the units, coordination with other units, guidelines, processes and protocols used, participation and communication with patients and their families, teaching activity, and research linked to the units.

**Conclusions:**

This project aimed to help understand the general situation of AIPUs in Spain and its different Regions, contribute to enhancing the benchmarking and harmonization among Spanish Regions, and provide data for future comparisons with other countries.

**International Registered Report Identifier (IRRID):**

RR1-10.2196/26214

## Introduction

In Spain, as a consequence of the decentralization set out in the Spanish Constitution of 1978, the jurisdiction for health care provision was transferred to each of the 17 Regions in Spain (known as Autonomous Communities) to constitute regional health care services. These act as the administrative managerial structure that integrates all the health centers, services, and establishments of each Region, as well as the provincial and city councils and any other interregional territorial administration.

Thus, since 1981, health care functions and services have been transferred from the central government to the different Spanish Regions. The first Region to have these functions transferred was Catalonia in 1981, followed by Andalucía in 1984, and eventually the Region of Madrid, where the transfer process was finalized in 2002. As a result, despite sharing a model and common guideline, there are differences both in terms of structure as well as the way in which the different health care systems function within each Region.

This decentralization of health care services over 2 decades has resulted in the coexistence of different models within the Public Health Care System, despite the fact that the Spanish General Health Law defines common guidelines. Thus, in practice, the transfer process has introduced its own characteristics and differential traits in the implementation of this law in each Region. In this regard, in 1987, the Interterritorial Board of the Spanish National Health Care System was created, to represent and coordinate the services provided by all the Regional Health Care Services. This Board was defined as the “permanent body of coordination, cooperation, communication, and information of Health Services” between each other and with the State Administration, with the aim of “promot[ing] the cohesion of the [Spanish] Health System through an effective guarantee of the rights of citizens throughout the country” [1].

The specialty of psychiatry has not been unaffected by these changes, and currently there are inter-Regional differences in the structure and care of psychiatric patients. This fact is understood by psychiatry professionals in Spain, who have stated in different forums and meetings that there is interest in generating a comprehensive overview of psychiatric care models and the performance of acute inpatient psychiatric units (AIPUs) in Spain, integrating the different operating models in the various Spanish Regions.

The present study was therefore proposed with the aim of understanding the current state of AIPUs in Spain through a scorecard that allows the current situation to be visualized in terms of resources, processes, and outputs. It will permit intra- and inter-Regional analysis of unmet needs, taking into consideration the idiosyncrasies of each Region. Moreover, by comparing the strengths and weaknesses among Regions, benchmarking and therefore harmonization regarding Spanish AIPUs could be proposed to health care planners. Finally, the results of this study could provide data for future comparisons with other countries.

An AIPU is defined as the hospital unit where psychiatric treatments are administered that require short-term inpatient admission (in general, less than 1 month). Admission of a patient to an AIPU is indicated in situations where it is not possible to provide outpatient care for an acute psychiatric process or for the exacerbation of a chronic process [2]. In line with this, the 1983 Mental Health Act for England and Wales defines short hospital stays as those that are less than 28 days [3]. The World Health Organization Mental Health Atlas 2017 [4] defines psychiatric hospital rooms in general hospitals as units that provide hospital health care for the management of patients with acute mental problems and the period of stay is generally short (weeks to months).

## Methods

### Questionnaire

A Scientific Committee comprised of professionals who understand the functioning of the AIPU in the different representative Spanish Regions was appointed. A questionnaire consisting of 104 questions was designed to be answered online by the staff member responsible for the AIPU in the different Regions. As a basis for its design, a literature review was carried out to identify the main resources, processes, and results indicators for AIPUs in Spain and at the international level. The search in PubMed was performed using the terms: “acute inpatient psychiatric units” AND [“quality” OR “indicators” OR “mental health services” OR “measuring” OR “improvement”]. Indicators and scorecards for results monitoring and assessment included in the mental health plans of the different Spanish Regions, clinical management plans and activity reports of psychiatry services, and recommendations and specialized publications on management systems were also reviewed. Based on the results of this review [5-26], a first draft of the survey was drawn up using the scorecards and indicators of resources, processes, and results in AIPUs. Finally, this first draft was reviewed by the Scientific Committee, who selected what they considered to be the more important variables and indicators. The final questionnaire was included in the 123 Form Builder online survey platform [27]. The online version was also reviewed by the Scientific Committee to ensure that it was easy to understand and complete.

### Data Collection

The data collection period was open for more than 6 months, between July 2018 and January 2019. At beginning of this period, emails were sent out to all the designated heads of the AIPUs to introduce the study and to provide access to a link to the questionnaire. In order to obtain the most complete responses possible, both in terms of the participating centers as well as the percentage of questions answered per questionnaire, a total of 10 general reminders were sent out via email to the units that had not started or completed the questionnaire. After 4 weeks, personalized emails were sent out to the person in charge of the unit whenever a response was not obtained.

The online questionnaire sent out to the inpatient units of the different Spanish Regions was divided into 11 sections, including data on the resources, processes, and results of the AIPU, plus a General section asking for general and complementary information about the AIPU*, *with the aim of collecting, in a structured manner, the most complete set of information on the units (see Table 1). 

**Table 1:** Survey sections and number of questions.

Section	Description	Number of questions
1. Number of beds	The number of beds available per 100,000 inhabitants	2
2. Multidisciplinary team of the AIPU^a^	The number of staff available in each AIPU	9
3. Infrastructure and AIPU models	The architectural structure, resources and the AIPU model: locked or nonlocked	19
4. Profiles of patients admitted to AIPUs	Age, patients over 65 years old, patients with cognitive impairment, and patients under 18 years old	8
5. Access Organization of Care	The route of referral to the AIPU, patient distribution, use of scales, questionnaires, clinical guidelines, and standardized operating procedures	14
6. Specific interventions offered by the AIPU	Specific interventions such as: medical interdisciplinary consultations, electroconvulsive therapy, or transcranial magnetic stimulation	5
7. Integration and coordination of the AIPU	The integration and coordination between the professionals that make up the AIPU and also with other hospital services, as well as other levels of care	1
8. Participation of people with mental health problems and their relatives	The participation of the patients and their relatives in the decision processes and the use of satisfaction questionnaires for patients and relatives	5
9. Activity of the AIPU	Markers of activity such as total admissions per 100,000 inhabitants, ratio of total planned vs emergency admissions, ratio of voluntary vs nonvoluntary admissions, percentage of admissions per diagnostic group, mean stay, readmissions (at 7 and 30 days), voluntary discharges	6
10. Training	Number of staff taking part in teaching activities, as well as the number of medical and nursing students	3
11. Research	The participation of the AIPU in public and private research, development, and innovation	4
General and complementary information	Hospital type, participant profile, quality indicators, and best practices	28

^a^AIPU: acute inpatient psychiatric unit.

The survey included a combination of dichotomous (yes/no) and multiple-choice questions, questions asking for specific data or figures, and open questions requiring free-text responses. The results of the survey are presented as mean and standard deviation for quantitative results and as percentages in the case of qualitative variables. Data for Spain as a whole and for each Spanish Region are presented.

### Variables Collected Through the Questionnaire

#### AIPU Resources

The number of beds available per 100,000 inhabitants was collected [5]. To assess the multidisciplinary team of the AIPU [10], the number of staff available in each AIPU to attend to patients with mental health problems during their hospital stay was analyzed. The teams that make up the AIPU were usually professionals from psychiatry, nursing, psychology, social work, and occupational therapy. Full-time equivalent data were studied, corresponding to full-time staff.

Regarding the infrastructure and AIPU models [22], the architectural structure was studied: availability of individual or shared rooms; square meterage; and availability of meeting rooms, cafeterias, and visiting room. The AIPU model was also collected: locked or nonlocked units.

#### Processes and Outputs

To obtain the profiles of patients admitted to AIPUs [25], data were collected on patient age, percentage of patients over 65 years old, percentage of patients with cognitive impairment, and the percentage of patients under 18 years old.

Regarding access and organization of care [16,17], the following were analyzed: the route of referral to the AIPU (emergency services, mental health center, other services, outpatient clinics, primary care, other), criteria used by AIPU staff to distribute patients who are admitted, use of validated scales and questionnaires in the patient assessment, use of clinical guidelines, and application of standardized operating procedures (such as suicide risk prevention, immobilization, inpatient admission process, electroconvulsive therapy, prevention of unplanned departure from inpatient care, treatment with clozapine).

In addition to the treatment of inpatients, we collected information on the specific interventions offered by the AIPU [13-15]: medical interdisciplinary consultations for hospitalized patients, electroconvulsive therapy, and transcranial magnetic stimulation. Data on access to different pharmacological options for the control of agitation were also collected, as well as access to different extended-release antipsychotic drugs for treatment during hospitalization.

The integration and coordination between the professionals that make up the AIPU [11,20], as well as the possible forms of communication, were studied (face-to-face meetings, clinical sessions, telephone, email, electronic health records, videoconference, others). Information was noted on the coordination with other hospital services (eg, internal medicine, neurology, anesthesiology), as well as other levels of care, such as mental health centers, medium-to-long stay units, outpatient hospitals, drug-dependency care networks, and primary care. Finally, data on referrals at discharge were also collected.

The existence of official tools for the participation of the relatives or guardians of patients with mental health issues admitted to the AIPU [6-9], such as personal interviews, informed consent, and written material specifically for good communication with relatives or guardians as well as the patient, was collected. Data on the use of satisfaction questionnaires for patients and relatives were also compiled.

The following activity markers of the AIPU [16-19,21-25] were collected: total admissions to AIPU per 100,000 inhabitants, ratio of total planned vs emergency admissions, ratio of voluntary vs nonvoluntary admissions, percentage of admissions per diagnostic group, mean length of stay, adjusted mean stay index, readmissions to the AIPU (at 7 and 30 days), and voluntary discharges.

Regarding training [17,19,25], data on the number of staff, as well as the number of medical and nursing students, taking part in teaching activities were collected.

To assess research [17,19,25], participation of the AIPU in Research, Development and Innovation (R&D&i) projects and the type of funding were analyzed. We also examined the number of staff from the AIPU who are integrated in established research structures such as the Carlos III Health Institute, Biomedical Research Networking Center for Mental Health Network (CIBERSAM), and Network of Research on Addictive Disorders (Red de Investigación en Trastornos Adictivos). Finally, the different lines of research carried out by the AIPU were collected.

## Results

**Figure 1:**
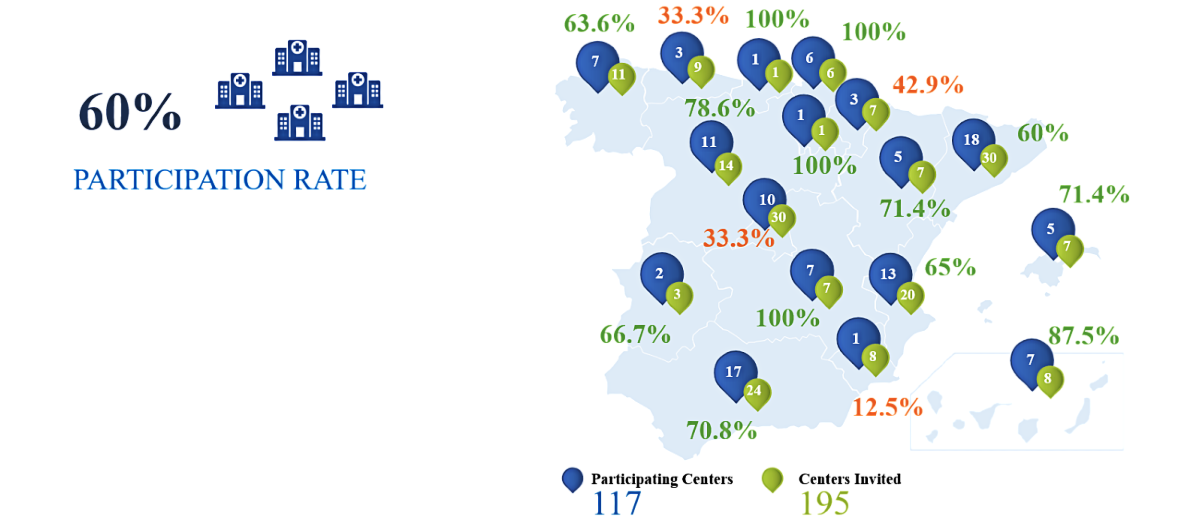
Distribution of the contacted acute inpatient psychiatric units (AIPUs) and participation rates per Region.

A total of 195 AIPUs were identified in the 17 regions. The designated person in charge of each unit was informed and invited to participate in the study. Finally, information was obtained from 117 AIPUs throughout Spain, providing relevant data on resources and activity; this indicates participation by 60.0% of the identified units. This general response rate, at a 95% confidence level, had a margin of error or confidence interval of 6%. A representation of units from all Regions was achieved (see Figure 1).

In most cases, the contact and request for participation were made through the heads of psychiatry services or departments and coordinators of the AIPU; in 70.8% (83/117) of cases, the person who filled out the questionnaire was the coordinator of the AIPU.

**Figure 2:**
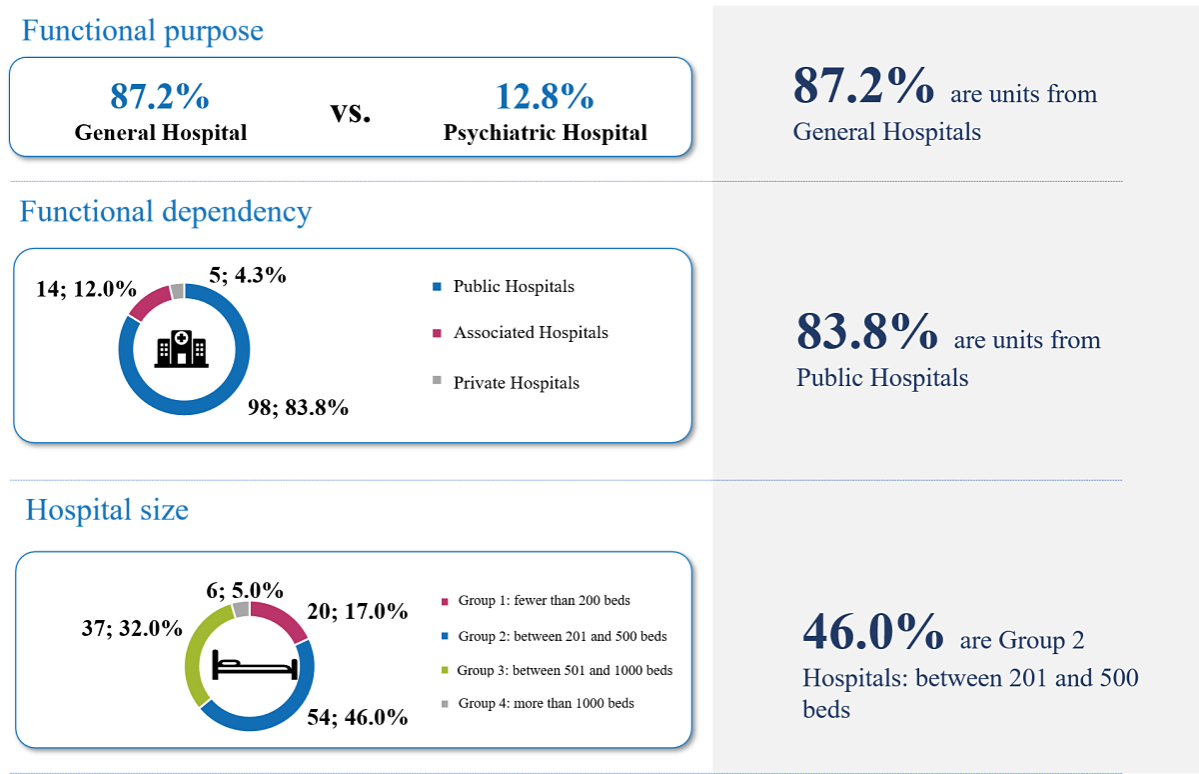
Profile of the units that completed the acute inpatient psychiatric unit (AIPU) questionnaire.

According to the functional purpose of the hospital where they are located, 87.2% (102/117) of the participating AIPUs were located in general hospitals, while 12.8% (15/117) belonged to psychiatric hospitals. In terms of the functional dependency of the center, 83.8% (98/117) were in public hospitals. Last, with regards to the hospital size, it should be noted that, although the majority of participating units (54/117, 46%) were located in medium-size hospitals (from 201 to 500 beds, known as group 2 in Spain), the questionnaire was completed by units of hospitals with less than 200 beds (group 1), accounting for 17% (20/117) of all units, and units belonging to hospitals with 500-1000 beds (group 3), accounting for 32% (37/117) of all units. Only 5% (6/117) of the participating units were found in hospitals with more than 1000 beds (group 4; see Figure 2).

## Discussion

The aim of this study was to understand the current state of AIPUs in Spain through a scorecard that allows the current situation to be visualized in terms of resources, processes, and outputs in the different Spanish Regions. It will permit intra- and inter-Regional analysis and could provide data for future comparisons with other countries.

We estimate that 60.0% (117/195) of all Spanish AIPUs provided data to this study, which means that the results obtained in the different areas studied are quite representative of the reality of the situation of these units in Spain. This is also supported by the participation of AIPUs from all Regions, as well as a high level of participation of AIPUs from public centers, both general and specialized hospitals, and the participation of hospitals with different numbers of beds.

These findings will allow us to present the ways in which AIPUs in Spain function and to examine the structural and functional differences among the different Spanish Regions (and could also allow comparison with other countries). Analysis of these results will allow areas of improvement to be identified for which possible actions may be designed in response and whose implementation will have a positive impact on the working of the AIPU. At the same time, it will also enable us to lay down the foundations for a continuous monitoring system that will allow the measurement of said impact. Accordingly, we hope that our findings will enable us to draw conclusions to help us achieve excellence in the planning of health care for mental health patients and their families and friends.

## Abbreviations

AIPU: acute inpatient psychiatric unit

## References

[1] (2003). Act 16/2003 on the Cohesion and Quality of the National Health System. Ministerio de Sanidad.

[2] Sistema de Información de Atención Primaria – SIAP. Ministerio de Sanidad.

[3] (2021). The Mental Health Act 1983. Rethink Mental Illness.

[4] (2018). Mental Health ATLAS 2017. World Health Organization.

[5] (2021). Global Health Observatory Data Repository: Beds. World Health Organization.

[6] Proyecto V (2018). Necesidades de las personas con esquizofrenia/psicosis y sus cuidadores. Confederación Salud Mental España.

[7] Bird V, Miglietta E, Giacco D, Bauer M, Greenberg L, Lorant V, Moskalewicz J, Nicaise P, Pfennig A, Ruggeri M, Welbel M, Priebe S (2019). Factors associated with satisfaction of inpatient psychiatric care: a cross country comparison. Psychol. Med.

[8] Sartorius N, Sanz J, Pérez R (2006). Cuestionarios de satisfacción en psiquiatría: Ventajas y controversias. Rev. Asoc. Esp. Neuropsiq.

[9] de los Cobos JP, Valero S, Haro G, Fidel G, Escuder G, Trujols J, Valderrama JC (2002). Development and psychometric properties of the Verona Service Satisfaction Scale for methadone-treated opioid-dependent patients (VSSS-MT). Drug and Alcohol Dependence.

[10] Giacco D, Bird V, Ahmad T, Bauer M, Lasalvia A, Lorant V, Miglietta E, Moskalewicz J, Nicaise P, Pfennig A, Welbel M, Priebe S (2018). The same or different psychiatrists for in- and out-patient treatment? A multi-country natural experiment. Epidemiol Psychiatr Sci.

[11] Arango C, Bernardo M, Bonet P, Cabrera A, Crespo-Facorro B, Cuesta MJ, González Nel, Parrabera S, Sanjuan J, Serrano A, Vieta E, Lennox BR, Melau M (2017). When the healthcare does not follow the evidence: The case of the lack of early intervention programs for psychosis in Spain. Rev Psiquiatr Salud Ment.

[12] Salvador-Carulla L, Salinas JA, Martín M, Grané M, Gibert K, Roca M, Bulbena A (2010). Indicadores para la evaluación de sistemas de salud mental en España. Grupo de Trabajo de Gestión Clínica de la Sociedad Española de Psiquiatría.

[13] Lehman A, Lieberman J, Dixon L, McGlashan TH, Miller AL, Perkins DO, Kreyenbuhl J, American Psychiatric Association, Steering Committee on Practice Guidelines (2004). Practice guideline for the treatment of patients with schizophrenia, second edition. Am J Psychiatry.

[14] Gaebel W, Weinmann S, Sartorius N, Rutz W, McIntyre JS (2005). Schizophrenia practice guidelines: international survey and comparison. Br J Psychiatry.

[15] (2012). Guideline on clinical investigation of medicinal products, including depot preparations in the treatment of schizophrenia. European Medicines Agency.

[16] (2007). Estrategia en Salud Mental del Sistema Nacional de Salud. Ministerio de Sanidad y Consumo.

[17] López-Ibor J, Reneses B (2012). La gestión clínica y la gestión de procesos en el ámbito de los trastornos mentales. Actas Esp Psiquiatr.

[18] Killaspy H, Cardoso G, White S, Wright C, Caldas de Almeida JM, Turton P, Taylor TL, Schützwohl M, Schuster M, Cervilla JA, Brangier P, Raboch J, Kalisova L, Onchev G, Alexiev S, Mezzina R, Ridente P, Wiersma D, Visser E, Kiejna A, Adamowski T, Ploumpidis D, Gonidakis F, King M (2016). Quality of care and its determinants in longer term mental health facilities across Europe; a cross-sectional analysis. BMC Psychiatry.

[19] Perlman CM, Hirdes JP, Barbaree H, Fries BE, McKillop I, Morris JN, Rabinowitz T (2013). Development of mental health quality indicators (MHQIs) for inpatient psychiatry based on the interRAI mental health assessment. BMC Health Serv Res.

[20] (2014). Transversalidad y continuidad asistencial en salud mental. Ministerio de Sanidad, Servicio sociales e Igualdad.

[21] Nicolucci A, Greenfield S, Mattke S (2006). Selecting indicators for the quality of diabetes care at the health systems level in OECD countries. Int J Qual Health Care.

[22] Wobrock T, Weinmann S, Falkai P, Gaebel W (2009). Quality assurance in psychiatry: quality indicators and guideline implementation. Eur Arch Psychiatry Clin Neurosci.

[23] Jiménez RE, Lam RM, Marot M, Delgado A (2004). Observed-predicted length of stay for an acute psychiatric department, as an indicator of inpatient care inefficiencies. Retrospective case-series study. BMC Health Serv Res.

[24] Al-Sughayir MA (2016). Effect of accreditation on length of stay in psychiatric inpatients: pre-post accreditation medical record comparison. Int J Ment Health Syst.

[25] Längle G, Baum W, Wollinger A, Renner G, U'Ren R, Schwärzler F, Eschweiler GW (2003). Indicators of quality of in-patient psychiatric treatment: the patients' view. Int J Qual Health Care.

[26] Bernardo M, de Dios C, Pérez V, Ignacio E, Serrano M, Vieta E, Mira JJ, Guilabert M, Roca M (2018). Quality indicators in the treatment of patients with depression, bipolar disorder or schizophrenia. Consensus study. Rev Psiquiatr Salud Ment.

[27] (2021). 123FormBuilder.

